# Central and local arterial stiffness in White Europeans compared to age-, sex-, and BMI-matched South Asians

**DOI:** 10.1371/journal.pone.0290118

**Published:** 2023-08-24

**Authors:** Koen M. van der Sluijs, Jos Thannhauser, Iris M. Visser, P. M. Nabeel, Kiran V. Raj, Afrah E. F. Malik, Koen D. Reesink, Thijs M. H. Eijsvogels, Esmée A. Bakker, Prabhdeep Kaur, Jayaraj Joseph, Dick H. J. Thijssen

**Affiliations:** 1 Department of Medical BioSciences, Radboud University Medical Center, Nijmegen, Gelderland, The Netherlands; 2 Faculty of Science and Technology, Department of Cardiovascular and Respiratory Physiology, University of Twente, Enschede, Overijssel, The Netherlands; 3 Technical Medicine, University of Twente, Enschede, Overijssel, The Netherlands; 4 Healthcare Technology Innovation Centre, Indian Institute of Technology Madras, Chennai, Tamil Nadu, India; 5 Department of Electrical Engineering, Indian Institute of Technology Madras, Chennai, Tamil Nadu, India; 6 Department of Biomedical Engineering, CARIM School for Cardiovascular Diseases, Maastricht University Medical Center, Maastricht, Limburg, The Netherlands; 7 National Institute of Epidemiology, Indian Council of Medical Research, Chennai, Tamil Nadu, India; University of Perugia, ITALY

## Abstract

**Background:**

Ethnicity impacts cardiovascular disease (CVD) risk, and South Asians demonstrate a higher risk than White Europeans. Arterial stiffness is known to contribute to CVD, and differences in arterial stiffness between ethnicities could explain the disparity in CVD risk. We compared central and local arterial stiffness between White Europeans and South Asians and investigated which factors are associated with arterial stiffness.

**Methods:**

Data were collected from cohorts of White Europeans (the Netherlands) and South Asians (India). We matched cohorts on individual level using age, sex, and body mass index (BMI). Arterial stiffness was measured with ARTSENS® Plus. Central stiffness was expressed as carotid-femoral pulse wave velocity (cf-PWV, m/s), and local carotid stiffness was quantified using the carotid stiffness index (Beta) and pressure-strain elastic modulus (Epsilon, kPa). We compared arterial stiffness between cohorts and used multivariable linear regression to identify factors related to stiffness.

**Results:**

We included n = 121 participants per cohort (age 53±10 years, 55% male, BMI 24 kg/m^2^). Cf-PWV was lower in White Europeans compared to South Asians (6.8±1.9 vs. 8.2±1.8 m/s, p<0.001), but no differences were found for local stiffness parameters Beta (5.4±2.4 vs. 5.8±2.3, p = 0.17) and Epsilon (72±35 vs. 70±31 kPa, p = 0.56). Age (standardized β, 95% confidence interval: 0.28, 0.17–0.39), systolic blood pressure (0.32, 0.21–0.43), and South Asian ethnicity (0.46, 0.35–0.57) were associated with cf-PWV; associations were similar between cohorts (p>0.05 for interaction). Systolic blood pressure was associated with carotid stiffness in both cohorts, whereas age was associated to carotid stiffness only in South Asians and BMI only in White Europeans.

**Conclusion:**

Ethnicity is associated with central but not local arterial stiffness. Conversely, ethnicity seems to modify associations between CVD risk factors and local but not central arterial stiffness. This suggests that ethnicity interacts with arterial stiffness measures and the association of these measures with CVD risk factors.

## Introduction

Cardiovascular disease (CVD) is the world’s leading cause of morbidity and mortality [1, 2]. The prevalence of CVD is known to vary across different ethnic groups [3, 4]. South Asians demonstrate a higher burden of conventional CVD risk factors than White populations [5–8]. Traditional CVD risk factors (e.g., obesity, type 2 diabetes mellitus, dyslipidemia, hypertension, and tobacco use) and CVD events present themselves at a younger age [7–11], and contemporary risk prediction models underestimate CVD risk in South Asians [12–14]. Possibly, other factors play a role in CVD development in South Asians, which may be reflected by a distinct impact of ethnicity on vascular health [3, 10, 15].

Central arterial stiffness, quantified by carotid-femoral pulse wave velocity (cf-PWV), is an early marker of impaired vascular health [16, 17]. Cf-PWV is known to predict cardiovascular events and all-cause mortality [17–22]. Previous work evaluated ethnicity-based differences in cf-PWV to better understand disparities in CVD risk between South Asian and White populations [3, 23–28]. These studies demonstrated inconsistent findings, with cf-PWV reported to be lower [3], not different [23–25], or higher [26–28] in South Asians compared to White populations. In addition to central arterial stiffness, studies have explored local carotid stiffness, such as carotid distensibility and elasticity. Although studies reported ethnicity-based differences in local stiffness [29–32], previous work did not include South Asians. A comprehensive evaluation of central and local stiffness, combined with matching of participants at individual level, may provide better insight into the impact of ethnicity.

To this extent, we first compared central and local arterial stiffness between White Europeans from the Netherlands and South Asians from India. Second, we identified factors associated with central and local arterial stiffness and evaluated whether these factors differ between the populations to better understand the impact of ethnicity on CVD risk.

## Materials and methods

### Study populations

Data from the White European cohort were collected as part of an ongoing Dutch prospective cohort study (Nijmegen Exercise Study). Adult volunteers were recruited in May 2021, and n = 265 individuals were included between May and September 2021. Ethical approval was obtained from the local Medical Research Ethics Committee (NL36743.091.11).

Data from the South Asian cohort were derived from an Indian cross-sectional study (n = 1,074) executed in the Tiruvallur district, Tamil Nadu, South India. Volunteers (age ≥30 years) were recruited and included between August 2017 and August 2018. Ethical approval was obtained from the Institutional Human Ethics Committee (NIE/IHEC/201407-02). Both studies were carried out in accordance with the Declaration of Helsinki, and all participants provided written informed consent. Additional information regarding the ethical, cultural, and scientific considerations specific to inclusivity in global research is included in the Supporting Information (S2 Checklist).

We performed one-to-one matching of the populations based on sex, age, and body mass index (BMI). We performed exact matching on sex and accepted a maximum age difference of 5 years and a maximum BMI difference of 2.5 kg/m^2^.

### Data collection

General patient characteristics were derived from questionnaires, including age, sex, tobacco use, and medical history [33, 34]. As for medical history, we inquired the presence of physician-made diagnoses of diabetes mellitus, hypertension, hypercholesterolemia, and history of CVD events, including myocardial infarction, stroke, thrombosis, heart failure, and cardiopulmonary resuscitation.

Prior to the study procedures, participants were instructed to fast (White Europeans: 4 hours; South Asians: 10 hours) and to abstain from alcohol and caffeinated drinks for at least 18 hours, in line with the ARTERY society guidelines for the assessment of arterial pulse wave velocity [35]. Height (cm), weight (kg), and waist and hip circumferences (cm) were measured (Seca GmbH & Co. KG, Hamburg, Germany), from which body mass index (BMI, kg/m^2^) and waist-to-hip ratio (WHR) were calculated. Venous serum was used to analyze total cholesterol, high-density lipoprotein (HDL) cholesterol, low-density lipoprotein (LDL) cholesterol, and glucose (all in mmol/L).

### Assessment of arterial stiffness

Arterial stiffness measurements were performed with ARTSENS® Plus (Healthcare Technology Innovation Centre, Indian Institute of Technology Madras, Chennai, India), a recently developed and validated, noninvasive, image-free ultrasound device to assess central and local arterial stiffness [36–38]. The ARTSENS® Plus uses a single-element ultrasound probe and a femoral pressure cuff for the simultaneous recording of carotid diameter and femoral pressure pulse waveforms, from which real-time arterial stiffness parameters are evaluated. The A-mode data captured by the ultrasound probe are processed with validated automated algorithms to yield carotid diameter [39, 40].

After a 5-minute resting period in supine position, patient characteristics (age, sex, height, and weight) were recorded in the ARTSENS® Plus. Dedicated blood pressure cuffs were attached around the participant’s left upper arm and left thigh (widths of 12.5 cm and 14.0 cm, respectively), ensuring a tight and fixed fit. Noninvasive brachial systolic blood pressure (SBP), diastolic blood pressure (DBP), and heart rate were measured once by a medical-grade blood pressure monitor integrated in the ARTSENS® Plus. Three straight distances (mm) were measured with a tape measure to estimate the effective path length (*D*) travelled by the pulse wave, according to established methods [35, 41, 42]. The first distance (*i*) was measured between the sternal notch and the left common carotid artery site as identified by palpation. The second distance (*ii*) was measured between the sternal notch and the top of the thigh cuff. The third distance (*iii*) was measured between the top of the thigh cuff and the groin area where the femoral artery could be palpated. The three distances were recorded in the ARTSENS® Plus, which then estimated the effective path length as: *D = ii−i−iii*.

Next, the operator positioned the gel-covered ultrasound probe at the left common carotid artery site that was approximated earlier. Guided by the display of the A-mode ultrasound signal, the operator reoriented the probe such that strong and distinct echoes of the arterial walls were visible, allowing the software to recognize them and track the artery distension waveform. Simultaneously, the thigh cuff automatically inflated to sub-diastolic pressure to capture the femoral artery pressure waveforms. The measurement was completed after capturing ten synchronously-measured high-quality carotid artery distension cycles and femoral artery pressure waveforms. The pulse transit time was defined as the time delay between the feet of the carotid and femoral artery waveforms and was averaged over the ten cycles [38].

Measurements were performed by trained operators (White Europeans: single measurement by one of five operators; South Asians: duplicate measurement by two operators and average values used for analysis). The primary outcome measures were cf-PWV (m/s) for central arterial stiffness and carotid stiffness index (Beta) and pressure-strain elastic modulus (Epsilon, kPa) for local carotid stiffness.

### Statistics

Data were pseudonymized; only the authors involved in the data management procedures had access to information that could identify individual participants. Statistical analyses were performed with IBM SPSS Statistics for Windows, version 27 (IBM Corp., Armonk, N.Y., USA). For all analyses, p<0.05 was considered statistically significant. The figures presented in this article were created with RStudio (version 4.1.3) using the packages ggplot2 and forestplot. Continuous variables were visually inspected for Gaussian distribution using histograms, reported as means ± standard deviations and compared between the cohorts using independent sample t-tests. Categorical variables were reported as numbers (%) and compared between the cohorts using Fisher’s exact test.

Univariable linear regression analyses were performed with cf-PWV as a dependent variable and ethnicity, age, sex, BMI, hypertension, history of CVD event, tobacco use, SBP, DBP, total cholesterol, HDL cholesterol, LDL cholesterol, total-to-HDL cholesterol ratio, and glucose as independent variables. We only selected variables for which data were available in at least 80% of the participants of each cohort, and we only selected categorical variables with a minimum of five observations per category. Second, we used forward-stepwise entry to create a multivariable linear regression model of main effects. Only variables with p<0.1 in the univariable regression analyses were offered for stepwise entry. Visual inspection of the residuals was performed to check linearity. Multicollinearity of the variables included in the model was assessed using the variance inflation factor. We expanded the model by entering interaction terms between ethnicity and the independent variables included in the model to evaluate ethnicity-based differences. In case of a significant interaction term, we performed stratified analyses per cohort. Similar procedures were performed for the local arterial stiffness parameters, i.e. Beta and Epsilon.

## Results

A total of n = 242 participants were included, with n = 121 participants in both the White European and South Asian cohorts. Age was 53±10 years, and 67 (55%) participants were male in both groups. BMI was 24.1±3.3 and 23.9±3.7 kg/m^2^ in the White European and South Asian cohorts, respectively. Compared to White Europeans, South Asians were more often current smokers, had a higher WHR, and had higher glucose levels (Table 1). South Asians had lower SBP and DBP values and lower total and HDL cholesterol levels compared to White Europeans. LDL cholesterol level and prevalence of diabetes or hypercholesterolemia did not differ between cohorts (Table 1).

**Table 1:** Participant characteristics of the study cohorts.

	White Europeans	South Asians	p-value
	n = 121	n = 121	
**Matched variables**			
Age, yrs	53±10	53±10	0.93
Male sex	67 (55)	67 (55)	>0.99
Body mass index, kg/m^2^	24.1±3.3	23.9±3.7	0.66
**Cardiovascular risk factors**			
Diabetes mellitus *	1 (1)	5 (4)	0.21
Hypertension *	11 (9)	11 (9)	>0.99
Hypercholesterolemia ^¤^	9 (8)	3 (2)	0.08
History of CVD event *^,^^‡^	8 (7)	N/A	-
Tobacco use ^+^			<0.001
Current user	7 (6)	14 (12)	
Former user	33 (28)	6 (5)	
Never	79 (66)	101 (83)	
**Measurement characteristics**			
Height, cm	176±9	161±9	<0.001
Weight, kg	75±14	62±12	<0.001
Waist-to-hip ratio ^§^	0.87±0.08	0.97±0.04	<0.001
Systolic blood pressure, mmHg	132±15	121±21	<0.001
Diastolic blood pressure, mmHg	79±9	75±13	0.010
Total cholesterol, mmol/l ^¤^	5.2±0.9	4.8±1.1	0.002
HDL cholesterol, mmol/l ^¤^	1.6±0.4	1.2±0.3	<0.001
LDL cholesterol, mmol/l ^¤^	3.1±0.8	3.3±0.9	0.06
Total cholesterol/HDL ratio ^¤^	3.3±0.8	4.3±1.2	<0.001
Glucose, mmol/l ^¤^	4.9±0.5	5.8±1.3	<0.001

Abbreviations: CVD: cardiovascular disease, HDL: high-density lipoprotein, LDL: low-density lipoprotein, N/A: not available. Continuous variables are presented as means ± standard deviations; categorical variables are expressed as n (%).

* n = 118 for White European cohort.

^¤^ n = 117 for White European cohort.

^‡^ Includes myocardial infarction, stroke, thrombosis, heart failure, and cardiopulmonary resuscitation.

^+^ n = 119 for White European cohort.

^§^ n = 46 for White European cohort; n = 120 for South Asian cohort.

### Comparison of arterial stiffness between cohorts

**Fig 1:**
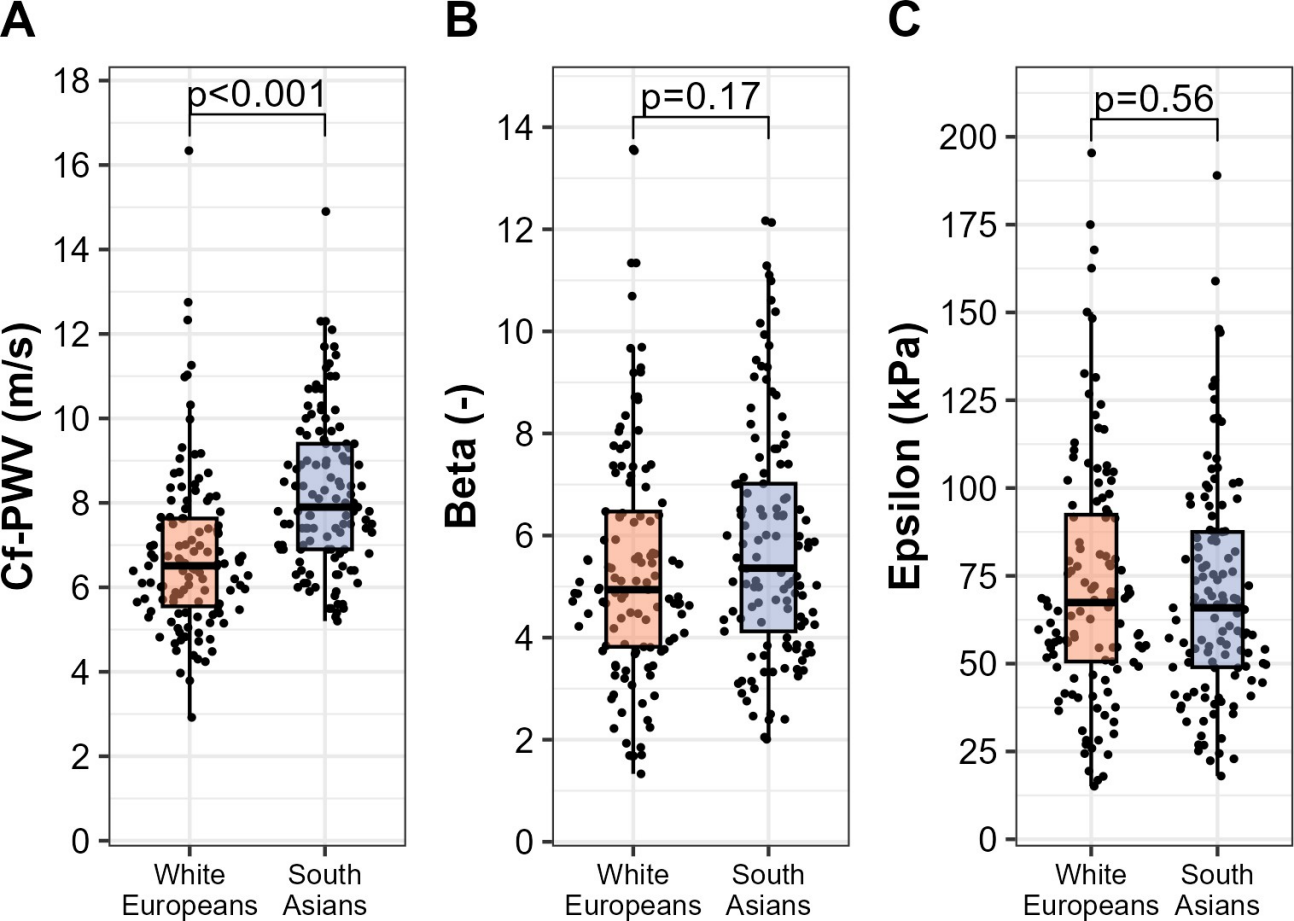
Central and local arterial stiffness for the White European (n = 121) and South Asian (n = 121) cohorts. A) Carotid-femoral pulse wave velocity (cf-PWV, m/s). B) Carotid stiffness index Beta (dimensionless). C) Pressure-strain elastic modulus Epsilon (kPa). Each dot represents an individual data point whereas box plots represent group statistics (Q1, median, Q3; whiskers extending up to 1.5 times the interquartile range). Differences between groups were assessed with an independent sample t-test.

Cf-PWV was significantly lower in the White European cohort compared to the South Asian cohort (6.8±1.9 vs. 8.2±1.8 m/s, p<0.001). No differences in local arterial stiffness were found between White Europeans and South Asians (Beta: 5.4±2.4 vs. 5.8±2.3, p = 0.17; Epsilon: 72±35 vs. 70±31 kPa, p = 0.56, respectively) (Fig 1).

### Factors associated with arterial stiffness

Univariable linear regression revealed significant associations of cf-PWV, Beta, and Epsilon with the independent variables (S1–S3 Tables). In all subsequent multivariable linear regression models, linearity was assumed based on visual inspection of residuals.

#### Cf-PWV

**Fig 2:**
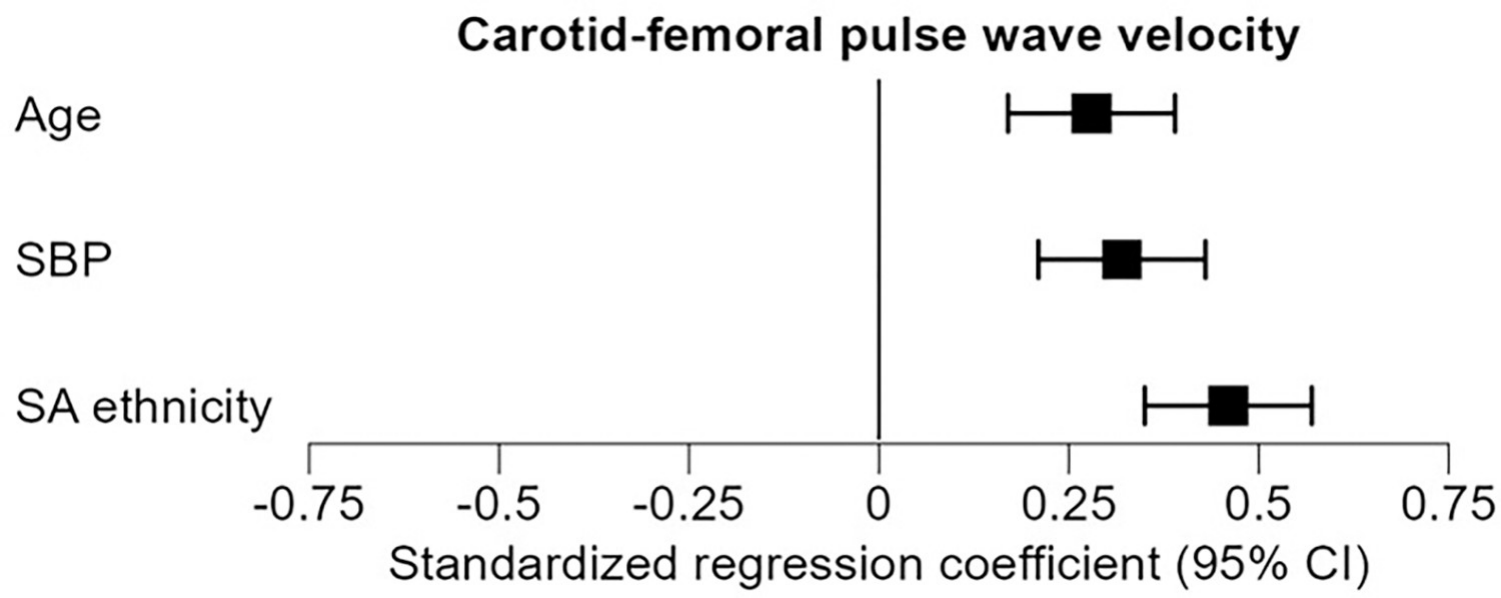
Associations with central arterial stiffness parameter carotid-femoral pulse wave velocity. Estimates of the standardized regression coefficients of the multivariable model are shown; error bars represent the 95% confidence intervals. Abbreviations: CI: confidence interval, SA: South Asian, SBP: systolic blood pressure.

The multivariable model without interaction terms with cf-PWV as dependent variable demonstrated associations with age (standardized coefficient β = 0.28; 95% confidence interval (CI): [0.17, 0.39]; p<0.001), SBP (β = 0.32; CI: [0.21, 0.43]; p<0.001), and South Asian ethnicity (β = 0.46; CI: [0.35, 0.57]; p<0.001) (Fig 2). The associations were not different between ethnicities (p>0.05 for interactions).

#### Beta

**Fig 3:**
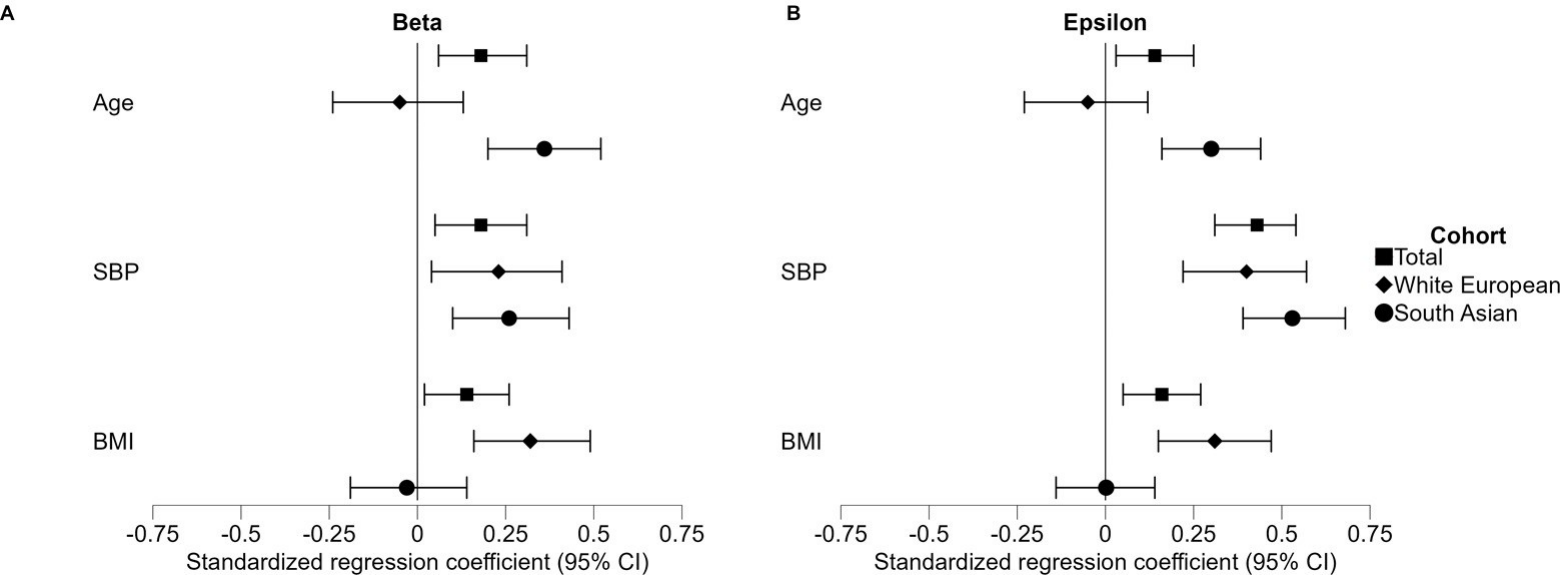
Associations with local arterial stiffness parameters A) carotid stiffness index Beta, and B) pressure-strain elastic modulus Epsilon. Estimates of the standardized regression coefficients of the multivariable model are shown; error bars represent the 95% confidence intervals. Abbreviations: BMI: body mass index, CI: confidence interval, SBP: systolic blood pressure.

The multivariable model for carotid stiffness index Beta showed associations with age (β = 0.18; CI: [0.06, 0.31]; p = 0.005), SBP (β = 0.18; CI: [0.05, 0.31]; p = 0.007), and BMI (β = 0.14; CI: [0.02, 0.26]; p = 0.026). Significant interaction terms were found between ethnicity and age and BMI, so separate models were created for the White European cohort and South Asian cohort. The stratified analysis revealed that BMI was associated with carotid stiffness index Beta in the White European cohort but not in the South Asian cohort. Moreover, age was significantly associated to carotid stiffness index Beta in the South Asian cohort but not in the White European cohort (Fig 3A).

#### Epsilon

In the multivariable model for pressure-strain elastic modulus Epsilon, we found associations with age (β = 0.14; CI: [0.03, 0.25]; p = 0.017), SBP (β = 0.43; CI: [0.31, 0.54]; p<0.001), and BMI (β = 0.16; CI: [0.05, 0.27]; p = 0.006). Because of significant interaction terms between ethnicity and age and BMI, we created cohort-specific models. The stratified analysis showed a significant association of Epsilon with BMI in the White European cohort and revealed that Epsilon was associated to age in the South Asian cohort (Fig 3B).

## Discussion

We compared central and local arterial stiffness between a White European and a matched South Asian cohort. We found a significantly lower cf-PWV in the White European cohort compared to the South Asian cohort, whilst no such differences were found for local arterial stiffness. Second, we identified factors associated with central and local arterial stiffness (e.g., age, SBP, and BMI), and we investigated whether these associations differed between ethnic cohorts. Central arterial stiffness was significantly associated with age and SBP, and we found that ethnicity did not alter this relation. In contrast, we found ethnicity-dependent associations for local arterial stiffness. Age was associated with local stiffness only in South Asians, and BMI was associated with local stiffness only in White Europeans, whilst SBP was associated with local stiffness in both cohorts. Altogether, our findings suggest that ethnicity is associated with central, but not local arterial stiffness. Furthermore, ethnicity seems to alter the impact of factors associated with local, but not central arterial stiffness. This supports the existing evidence that ethnicity interacts with measures of arterial stiffness and adds new insight into the influence of ethnicity on factors related to CVD risk.

Our finding of a higher cf-PWV in South Asians is consistent with some [26–28], but in contrast with other studies [3]. In line with our findings, two small studies based on United Kingdom cohorts found higher cf-PWV values in South Asians than in White Europeans [27, 28]. Similarly, a Dutch study reported a higher cf-PWV in South Asians than in White Europeans aged >40 years [26]. In contrast, a large American study found a lower cf-PWV in South Asians than in the resident White population [3]. Moreover, some studies found no differences between the two populations [23–25]. These aforementioned studies are methodologically similar to our present study pertaining to sample size (range 131–4,211 participants), use of validated methods for measuring cf-PWV, and adjustment for conventional CVD risk factors. A possible explanation for the discrepancy in findings is that cf-PWV may be influenced by ethnic-based differences that were not adjusted for, such as WHR, body surface area, or proinflammatory state [7]. One important difference between studies is that we included participants residing in the countries of their ethnic origin. Whilst this may have introduced a between-country bias, it allowed us to evaluate the impact of ethnicity on arterial stiffness in the original ethnic settings.

By investigating which factors are related to central arterial stiffness, we found that only higher age and SBP were positively associated with cf-PWV. The relations between cf-PWV and age and SBP have been described previously [17, 22, 26, 43]. For example, a systematic review of 77 studies reported that age and SBP are strongly related to cf-PWV [43]. Notably, this review also reported that other CVD risk factors are not or only modestly related to cf-PWV, an observation that matches our findings. We add the insight that the association between age and central stiffness was not ethnicity-dependent, which agrees with the American study [3] but disagrees with the Dutch study [26]. In the latter study, the positive relation between age and cf-PWV was significantly greater for the South Asian group than for the Dutch group, resulting in a greater cf-PWV difference between ethnicities at higher age. Furthermore, we found that cf-PWV was significantly associated with age, an effect that was similarly present in both ethnic groups. This suggests that ethnicity does not alter the impact of risk factors on measures of central arterial stiffness. Altogether, our findings corroborate previous work and suggest that factors related to central arterial stiffness are robust and do not differ in direction and/or magnitude between South Asian and White European ethnicities.

To our knowledge, this study is the first to compare local carotid stiffness between these two populations. In contrast to our findings on central arterial stiffness, we found no differences between the White European and South Asian cohorts in measures of local arterial stiffness, which is somewhat unexpected. However, it fits with the evidence that central and local arterial stiffness are distinct measures that cannot be used interchangeably [44]. As both central and local measures are known to predict incident cardiovascular events and all-cause mortality [19, 44], combining central and local arterial stiffness measures may provide the most predictive information on vascular health status.

Although local carotid stiffness did not differ between groups, we found age, SBP, and BMI to relate to local carotid stiffness. The relation between higher carotid stiffness and older age [45–47] as well as the association between carotid stiffness and BMI have been described before [48–50]. Interestingly, we found that ethnicity impacted the associations for both local stiffness parameters. Specifically, we found that BMI was related to carotid stiffness in White Europeans, whilst age was associated with carotid stiffness in South Asians. Possibly, the distinct associations of BMI with local stiffness between cohorts may relate to differences in body composition. South Asians tend to have more visceral adipose tissue and a higher fat percentage at a given BMI level compared to White Europeans [7, 51, 52], which seems to alter the relation between BMI and CVD risk [53, 54]. Differences in body composition between cohorts may therefore contribute to the differences we observed in the association of BMI with local stiffness. The difference between cohorts in the association of age with carotid stiffness may, at least in part, be related to the earlier onset of CVD in South Asians compared to White Europeans [7, 8, 11]. As our study includes relatively young participants, the relation between age and carotid stiffness may have developed in the South Asian cohort but not yet in the White European cohort. Taken together, our observations suggest that ethnicity does not alter local carotid stiffness per se, but it may affect the underlying factors related to local stiffness.

Strengths of our study are the use of the same validated device to assess central and local arterial stiffness in both cohorts [37, 38], the inclusion of a large group of participants, and the inclusion of participants residing in the country of their ethnic origin. However, some limitations are present. A potential limitation is a selection bias introduced by the matching procedure. From the 265 and 1,074 individuals in the Dutch and Indian cohorts, respectively, 121 participants were included per cohort after matching. However, participant characteristics indicated that these samples represent the source populations well, so we expect the potential selection bias to be minimal. Another limitation is that data on CVD history in the South Asian cohort were not collected. This prevented us from providing a complete overview of CVD risk profile of both cohorts and from investigating whether this introduced a bias in our results. However, we incorporated conventional cardiovascular risk factors (e.g., the presence of hypertension or hypercholesterolemia, smoking behavior, and blood pressure) in our analyses and found no important differences. Therefore, we expect that the lack of this information does not impact our main outcomes.

In conclusion, we found that central arterial stiffness was lower in White Europeans compared to South Asians, but ethnicity did not affect the associations of risk factors (e.g., age, systolic blood pressure) and central stiffness. In contrast, we found no direct impact of ethnicity on local measures of arterial stiffness, whereas ethnicity did alter the associations between risk factors and local arterial stiffness. Therefore, our work demonstrates that ethnicity is related to measures of arterial stiffness and to the associations between these measures and CVD risk factors. Further research is warranted to investigate whether these ethnicity-based differences in central and local arterial stiffness have consequences for the development of CVD-related outcomes.

## Supporting information

S1 Checklist STROBE statement. (DOCX)

S2 Checklist Inclusivity in global research questionnaire. (DOCX)

S1 Table Univariable regression coefficients for the associations with carotid-femoral pulse wave velocity. (DOCX)

S2 Table Univariable regression coefficients for the associations with carotid stiffness index Beta. (DOCX)

S3 Table Univariable regression coefficients for the associations with pressure-strain elastic modulus Epsilon. (DOCX)

S4 Table Pulse transit time and distances for calculating carotid-femoral pulse wave velocity, per cohort. (DOCX)

## References

[1] Roth GA, Mensah GA, Johnson CO, Addolorato G, Ammirati E, Baddour LM, et al. Global Burden of Cardiovascular Diseases and Risk Factors, 1990–2019: Update From the GBD 2019 Study. Journal of the American College of Cardiology. 2020;76(25):2982–3021. doi: 10.1016/j.jacc.2020.11.010 . 33309175 PMC7755038

[2] Libby P. The changing landscape of atherosclerosis. Nature. 2021;592(7855):524–33. doi: 10.1038/s41586-021-03392-8 33883728

[3] Gujral UP, Mehta A, Sher S, Uphoff I, Kumar S, Hayek SS, et al. Ethnic differences in subclinical vascular function in South Asians, Whites, and African Americans in the United States. Int J Cardiol Heart Vasc. 2020;30:100598. Epub 2020/08/15. doi: 10.1016/j.ijcha.2020.100598 ; PubMed Central PMCID: PMC7408720. 32793802 PMC7408720

[4] Gordon NP, Lin TY, Rau J, Lo JC. Aggregation of Asian-American subgroups masks meaningful differences in health and health risks among Asian ethnicities: an electronic health record based cohort study. BMC Public Health. 2019;19(1). doi: 10.1186/s12889-019-7683-3 31760942 PMC6876105

[5] Rana A, de Souza RJ, Kandasamy S, Lear SA, Anand SS. Cardiovascular risk among South Asians living in Canada: a systematic review and meta-analysis. CMAJ Open. 2014;2(3):E183–E91. doi: 10.9778/cmajo.20130064 . 25295238 PMC4183167

[6] Anand SS, Yusuf S, Vuksan V, Devanesen S, Teo KK, Montague PA, et al. Differences in risk factors, atherosclerosis, and cardiovascular disease between ethnic groups in Canada: the Study of Health Assessment and Risk in Ethnic groups (SHARE). The Lancet. 2000;356(9226):279–84. doi: 10.1016/S0140-6736(00)02502-2 11071182

[7] Volgman AS, Palaniappan LS, Aggarwal NT, Gupta M, Khandelwal A, Krishnan AV, et al. Atherosclerotic Cardiovascular Disease in South Asians in the United States: Epidemiology, Risk Factors, and Treatments: A Scientific Statement From the American Heart Association. Circulation. 2018;138(1):CIR.00000000000. doi: 10.1161/CIR.0000000000000580 29794080

[8] Joshi P, Islam S, Pais P, Reddy S, Dorairaj P, Kazmi K, et al. Risk factors for early myocardial infarction in South Asians compared with individuals in other countries. Jama. 2007;297(3):286–94. Epub 2007/01/18. doi: 10.1001/jama.297.3.286 . 17227980

[9] Prabhakaran D, Jeemon P, Roy A. Cardiovascular Diseases in India. Circulation. 2016;133(16):1605–20. doi: 10.1161/circulationaha.114.008729 27142605

[10] Kandula NR, Kanaya AM, Liu K, Lee JY, Herrington D, Hulley SB, et al. Association of 10‐Year and Lifetime Predicted Cardiovascular Disease Risk With Subclinical Atherosclerosis in South Asians: Findings From the Mediators of Atherosclerosis in South Asians Living in America (MASALA) Study. Journal of the American Heart Association. 2014;3(5):e001117–e. doi: 10.1161/JAHA.114.001117 25277669 PMC4323809

[11] Jose PO, Frank ATH, Kapphahn KI, Goldstein BA, Eggleston K, Hastings KG, et al. Cardiovascular Disease Mortality in Asian Americans. Journal of the American College of Cardiology. 2014;64(23):2486–94. doi: 10.1016/j.jacc.2014.08.048 25500233 PMC4274749

[12] Kanjilal S. Application of cardiovascular disease risk prediction models and the relevance of novel biomarkers to risk stratification in Asian Indians. Vascular Health and Risk Management. 2008;4(1):199–211. doi: 10.2147/vhrm.2008.04.01.199 18629375 PMC2464770

[13] Goff DC, Lloyd-Jones DM, Bennett G, Coady S, D’Agostino RB, Gibbons R, et al. 2013 ACC/AHA Guideline on the Assessment of Cardiovascular Risk. Journal of the American College of Cardiology. 2014;63(25):2935–59. doi: 10.1016/j.jacc.2013.11.005 24239921 PMC4700825

[14] Bansal M, Ranjan S, Kasliwal RR. Cardiovascular Risk Calculators and their Applicability to South Asians. Curr Diabetes Rev. 2021;17(9):e100120186497. doi: 10.2174/1573399816999201001204020 . 33023452

[15] Din JN, Ashman OA, Aftab SM, Jubb AW, Newby DE, Flapan AD. Increased arterial stiffness in healthy young South Asian men. Journal of Human Hypertension. 2006;20(2):163–5. doi: 10.1038/sj.jhh.1001961 16306999

[16] Wilkinson IB, Mäki-Petäjä KM, Mitchell GF. Uses of Arterial Stiffness in Clinical Practice. Arteriosclerosis, Thrombosis, and Vascular Biology. 2020;40(5):1063–7. doi: 10.1161/ATVBAHA.120.313130 32102569 PMC7737371

[17] Chirinos JA, Segers P, Hughes T, Townsend R. Large-Artery Stiffness in Health and Disease. Journal of the American College of Cardiology. 2019;74(9):1237–63. doi: 10.1016/j.jacc.2019.07.012 31466622 PMC6719727

[18] Ben-Shlomo Y, Spears M, Boustred C, May M, Anderson SG, Benjamin EJ, et al. Aortic Pulse Wave Velocity Improves Cardiovascular Event Prediction. Journal of the American College of Cardiology. 2014;63(7):636–46. doi: 10.1016/j.jacc.2013.09.063 24239664 PMC4401072

[19] Vlachopoulos C, Aznaouridis K, Stefanadis C. Prediction of Cardiovascular Events and All-Cause Mortality With Arterial Stiffness. Journal of the American College of Cardiology. 2010;55(13):1318–27. doi: 10.1016/j.jacc.2009.10.061 20338492

[20] Safar ME. Arterial stiffness as a risk factor for clinical hypertension. Nature Reviews Cardiology. 2018;15(2):97–105. doi: 10.1038/nrcardio.2017.155 29022570

[21] Mitchell GF, Hwang S-J, Vasan RS, Larson MG, Pencina MJ, Hamburg NM, et al. Arterial stiffness and cardiovascular events: the Framingham Heart Study. Circulation. 2010;121(4):505–11. Epub 2010/01/18. doi: 10.1161/CIRCULATIONAHA.109.886655 . 20083680 PMC2836717

[22] Townsend RR, Wilkinson IB, Schiffrin EL, Avolio AP, Chirinos JA, Cockcroft JR, et al. Recommendations for Improving and Standardizing Vascular Research on Arterial Stiffness. Hypertension. 2015;66(3):698–722. doi: 10.1161/hyp.0000000000000033 26160955 PMC4587661

[23] Cruickshank JK, Silva MJ, Molaodi OR, Enayat ZE, Cassidy A, Karamanos A, et al. Ethnic Differences in and Childhood Influences on Early Adult Pulse Wave Velocity: The Determinants of Adolescent, Now Young Adult, Social Wellbeing, and Health Longitudinal Study. Hypertension (Dallas, Tex: 1979). 2016;67(6):1133–41. Epub 2016/05/02. doi: 10.1161/HYPERTENSIONAHA.115.07079 . 27141061 PMC4861702

[24] Sluyter JD, Hughes AD, Thom SAM, Lowe A, Camargo CA Jr, Hametner B, et al. Arterial waveform parameters in a large, population-based sample of adults: relationships with ethnicity and lifestyle factors. Journal of Human Hypertension. 2017;31(5):305–12. doi: 10.1038/jhh.2016.78 28004730 PMC5383734

[25] Park CM, Tillin T, March K, Jones S, Whincup PH, Mayet J, et al. Adverse effect of diabetes and hyperglycaemia on arterial stiffness in Europeans, South Asians, and African Caribbeans in the SABRE study. Journal of Hypertension. 2016;34(2). doi: 10.1097/HJH.0000000000000789 26628109 PMC4841389

[26] Snijder MB, Stronks K, Agyemang C, Busschers WB, Peters RJ, van den Born B-JH. Ethnic differences in arterial stiffness the Helius study. International Journal of Cardiology. 2015;191:28–33. doi: 10.1016/j.ijcard.2015.04.234 25965592

[27] Rezai M-R, Wallace AM, Sattar N, Finn JD, Wu FCW, Cruickshank JK. Ethnic Differences in Aortic Pulse Wave Velocity Occur in the Descending Aorta and May Be Related to Vitamin D. Hypertension. 2011;58(2):247–53. doi: 10.1161/HYPERTENSIONAHA.111.174425 21670413

[28] Webb DR, Khunti K, Lacy P, Gray LJ, Mostafa S, Talbot D, et al. Conduit vessel stiffness in British south Asians of Indian descent relates to 25-hydroxyvitamin D status. Journal of Hypertension. 2012;30(8). doi: 10.1097/HJH.0b013e328354f385 22688263

[29] Blaha MJ, Budoff MJ, Rivera JJ, Katz R, O’Leary DH, Polak JF, et al. Relationship of Carotid Distensibility and Thoracic Aorta Calcification. Hypertension. 2009;54(6):1408–15. doi: 10.1161/hypertensionaha.109.138396 19805639 PMC4118641

[30] Markert MS, Della-Morte D, Cabral D, Roberts EL, Gardener H, Dong C, et al. Ethnic differences in carotid artery diameter and stiffness: The Northern Manhattan Study. Atherosclerosis. 2011;219(2):827–32. doi: 10.1016/j.atherosclerosis.2011.08.028 21906739 PMC3226921

[31] Din-Dzietham R. Arterial stiffness is greater in African Americans than in whites evidence from the Forsyth County, North Carolina, ARIC cohort. American Journal of Hypertension. 2004;17(4):304–13. doi: 10.1016/j.amjhyper.2003.12.004 15062883

[32] Stern R, Tattersall MC, Gepner AD, Korcarz CE, Kaufman J, Colangelo LA, et al. Sex Differences in Predictors of Longitudinal Changes in Carotid Artery Stiffness. Arteriosclerosis, Thrombosis, and Vascular Biology. 2015;35(2):478–84. doi: 10.1161/atvbaha.114.304870 25477347 PMC4304990

[33] Maessen MFH, Verbeek ALM, Bakker EA, Thompson PD, Hopman MTE, Eijsvogels TMH. Lifelong Exercise Patterns and Cardiovascular Health. Mayo Clinic Proceedings. 2016;91(6):745–54. doi: 10.1016/j.mayocp.2016.02.028 27140541

[34] Schoofs MCA, Bakker EA, De Vries F, Hartman YAW, Spoelder M, Thijssen DHJ, et al. Impact of Dutch COVID-19 restrictive policy measures on physical activity behavior and identification of correlates of physical activity changes: a cohort study. BMC Public Health. 2022;22(1). doi: 10.1186/s12889-022-12560-y 35062927 PMC8777413

[35] Wilkinson IB, McEniery CM, Schillaci G, Boutouyrie P, Segers P, Donald A, et al. ARTERY Society guidelines for validation of non-invasive haemodynamic measurement devices: Part 1, arterial pulse wave velocity. Artery Research. 2010;4(2):34. doi: 10.1016/j.artres.2010.03.001

[36] Joseph J, Kiran R, Nabeel PM, Shah MI, Bhaskar A, Ganesh C, et al. ARTSENS® Pen—portable easy-to-use device for carotid stiffness measurement: technology validation and clinical-utility assessment. Biomedical Physics & Engineering Express. 2020;6(2):025013. doi: 10.1088/2057-1976/ab74ff 33438639

[37] Joseph J, Nabeel PM, Rao SR, Venkatachalam R, Shah MI, Kaur P. Assessment of Carotid Arterial Stiffness in Community Settings With ARTSENS®. IEEE Journal of Translational Engineering in Health and Medicine. 2021;9:1–11. doi: 10.1109/jtehm.2020.3042386 33329943 PMC7732146

[38] Nabeel PM, Raj KV, Joseph J. Image-free ultrasound for local and regional vascular stiffness assessment: the ARTSENS Plus. Journal of Hypertension. 2022;40(8):1537–44. Epub 20220621. doi: 10.1097/HJH.0000000000003181 . 35730407

[39] Sahani AK, Joseph J, Sivaprakasam M. Evaluation of the algorithm for automatic identification of the common carotid artery in ARTSENS. Physiological Measurement. 2014;35(7):1299–317. doi: 10.1088/0967-3334/35/7/1299 24853913

[40] Sahani AK, Joseph J, Radhakrishnan R, Sivaprakasam M. Automatic Measurement of End-Diastolic Arterial Lumen Diameter in ARTSENS. Journal of Medical Devices. 2015;9(4). doi: 10.1115/1.4030873

[41] Wohlfahrt P, Krajčoviechová A, Seidlerová J, Mayer O, Bruthans J, Filipovský J, et al. Arterial stiffness parameters: How do they differ? Atherosclerosis. 2013;231(2):359–64. doi: 10.1016/j.atherosclerosis.2013.10.006 24267252

[42] Van Bortel LM, Laurent S, Boutouyrie P, Chowienczyk P, Cruickshank JK, De Backer T, et al. Expert consensus document on the measurement of aortic stiffness in daily practice using carotid-femoral pulse wave velocity. Journal of Hypertension. 2012;30(3):445–8. doi: 10.1097/HJH.0b013e32834fa8b0 22278144

[43] Cecelja M, Chowienczyk P. Dissociation of Aortic Pulse Wave Velocity With Risk Factors for Cardiovascular Disease Other Than Hypertension. Hypertension. 2009;54(6):1328–36. doi: 10.1161/HYPERTENSIONAHA.109.137653 19884567

[44] van Sloten TT, Schram MT, van den Hurk K, Dekker JM, Nijpels G, Henry RMA, et al. Local Stiffness of the Carotid and Femoral Artery Is Associated With Incident Cardiovascular Events and All-Cause Mortality: The Hoorn Study. Journal of the American College of Cardiology. 2014;63(17):1739–47. 10.1016/j.jacc.2013.12.041. doi: 10.1016/j.jacc.2013.12.041 24583306

[45] Vermeersch SJ, Rietzschel ER, De Buyzere ML, De Bacquer D, De Backer G, Van Bortel LM, et al. Age and gender related patterns in carotid-femoral PWV and carotid and femoral stiffness in a large healthy, middle-aged population. Journal of Hypertension. 2008;26(7). 10.1097/HJH.0b013e3282ffac00 18551018

[46] O’Rourke MF, Hashimoto J. Mechanical Factors in Arterial Aging: A Clinical Perspective. Journal of the American College of Cardiology. 2007;50(1):1–13. 10.1016/j.jacc.2006.12.050. doi: 10.1016/j.jacc.2006.12.050 17601538

[47] Gepner AD, Korcarz CE, Colangelo LA, Hom EK, Tattersall MC, Astor BC, et al. Longitudinal Effects of a Decade of Aging on Carotid Artery Stiffness. Stroke. 2014;45(1):48–53. doi: 10.1161/strokeaha.113.002649 24253542 PMC3888489

[48] Engelen L, Bossuyt J, Ferreira I, van Bortel LM, Reesink KD, Segers P, et al. Reference values for local arterial stiffness. Part A: carotid artery. Journal of Hypertension. 2015;33(10). 10.1097/HJH.0000000000000654 26431185

[49] Ferreira I, Van De Laar RJ, Prins MH, Twisk JW, Stehouwer CD. Carotid Stiffness in Young Adults: A Life-Course Analysis of its Early Determinants. Hypertension. 2012;59(1):54–61. doi: 10.1161/hypertensionaha.110.156109 22068867

[50] Zebekakis PE, Nawrot T, Thijs L, Balkestein EJ, Van Der Heijden-Spek J, Van Bortel LM, et al. Obesity is associated with increased arterial stiffness from adolescence until old age. Journal of Hypertension. 2005;23(10):1839–46. doi: 10.1097/01.hjh.0000179511.93889.e9 16148607

[51] Kohli S, Sniderman AD, Tchernof A, Lear SA. Ethnic-Specific Differences in Abdominal Subcutaneous Adipose Tissue Compartments. Obesity. 2010;18(11):2177–83. doi: 10.1038/oby.2010.94 20448537

[52] Lear SA, Humphries KH, Kohli S, Birmingham CL. The Use of BMI and Waist Circumference as Surrogates of Body Fat Differs by Ethnicity**. Obesity. 2007;15(11):2817–24. doi: 10.1038/oby.2007.334 18070773

[53] Rao G, Powell-Wiley TM, Ancheta I, Hairston K, Kirley K, Lear SA, et al. Identification of Obesity and Cardiovascular Risk in Ethnically and Racially Diverse Populations. Circulation. 2015;132(5):457–72. doi: 10.1161/cir.0000000000000223 26149446

[54] Perini W, van Valkengoed IGM, Snijder MB, Peters RJG, Kunst AE. The contribution of obesity to the population burden of high metabolic cardiovascular risk among different ethnic groups. The HELIUS study. Eur J Public Health. 2020;30(2):322–7. doi: 10.1093/eurpub/ckz190 . 32053154

