# Supplementary material for: Central and local arterial stiffness in White Europeans compared to age-, sex-, and BMI-matched South Asians
Source: PLoS One. 2023 Aug 24;18(8):e0290118. doi: 10.1371/journal.pone.0290118 (PMC10449187; doi:10.1371/journal.pone.0290118)
Supplement: S1 Table — (DOCX) [file pone.0290118.s003.docx]

**S1 Table. Univariable regression coefficients for the associations with carotid-femoral pulse wave velocity.**

|  | **Unstandardized B [95% CI]** | **Standardized β** | **p-value** |
| --- | --- | --- | --- |
| South Asian ethnicity | 1.444 [0.967, 1.921] | 0.359 | <0.001 |
| Age, yrs | 0.075 [0.051, 0.099] | 0.369 | <0.001 |
| Male sex | 0.213 [-0.301, 0.726] | 0.053 | 0.42 |
| Body mass index, kg/m^2^ | 0.061 [-0.011, 0.134] | 0.107 | 0.10 |
| Hypertension | 0.970 [0.091, 1.848] | 0.140 | 0.031 |
| History of CVD event | 0.408 [-0.999, 1.815] | 0.053 | 0.57 |
| Former tobacco user * | -0.196 [-0.890, 0.497] | -0.036 | 0.58 |
| Current tobacco user * | 0.626 [-0.277, 1.529] | 0.088 | 0.17 |
| Systolic blood pressure, mmHg | 0.027 [0.015, 0.040] | 0.263 | <0.001 |
| Diastolic blood pressure, mmHg | 0.043 [0.021, 0.065] | 0.238 | <0.001 |
| Total cholesterol, mmol/l | -0.029 [-0.285, 0.227] | -0.014 | 0.83 |
| HDL cholesterol, mmol/l | -0.907 [-1.549, -0.264] | -0.178 | 0.006 |
| LDL cholesterol, mmol/l | 0.273 [-0.019, 0.565] | 0.119 | 0.07 |
| Total cholesterol/HDL ratio | 0.328 [0.099, 0.557] | 0.181 | 0.005 |
| Glucose, mmol/l | 0.525 [0.292, 0.757] | 0.278 | <0.001 |

Abbreviations: CI: confidence interval, CVD: cardiovascular disease, HDL: high-density lipoprotein, LDL: low-density lipoprotein. * Reference: tobacco never used.
