# Supplementary material for: Central and local arterial stiffness in White Europeans compared to age-, sex-, and BMI-matched South Asians
Source: PLoS One. 2023 Aug 24;18(8):e0290118. doi: 10.1371/journal.pone.0290118 (PMC10449187; doi:10.1371/journal.pone.0290118)
Supplement: S2 Table — (DOCX) [file pone.0290118.s004.docx]

**S2 Table. Univariable regression coefficients for the associations with carotid stiffness index Beta.**

|  | **Unstandardized B [95% CI]** | **Standardized β** | **p-value** |
| --- | --- | --- | --- |
| South Asian ethnicity | 0.420 [-0.175, 1.014] | 0.089 | 0.17 |
| Age, yrs | 0.055 [0.026, 0.084] | 0.234 | <0.001 |
| Male sex | -0.232 [-0.831, 0.368] | -0.049 | 0.45 |
| Body mass index, kg/m^2^ | 0.112 [0.028, 0.196] | 0.167 | 0.009 |
| Hypertension | 1.131 [0.102, 2.160] | 0.139 | 0.031 |
| History of CVD event | -0.759 [-2.469, 0.952] | -0.081 | 0.38 |
| Former tobacco user * | -0.246 [-1.057, 0.565] | -0.039 | 0.55 |
| Current tobacco user * | -0.245 [-1.304, 0.814] | -0.030 | 0.65 |
| Systolic blood pressure, mmHg | 0.031 [0.016, 0.046] | 0.253 | <0.001 |
| Diastolic blood pressure, mmHg | 0.007 [-0.020. 0.033] | 0.033 | 0.62 |
| Total cholesterol, mmol/l | 0.186 [-0.112, 0.484] | 0.080 | 0.22 |
| HDL cholesterol, mmol/l | -0.285 [-1.045, 0.476] | -0.048 | 0.46 |
| LDL cholesterol, mmol/l | 0.320 [-0.021, 0.660] | 0.120 | 0.07 |
| Total cholesterol/HDL ratio | 0.238 [-0.032, 0.508] | 0.112 | 0.08 |
| Glucose, mmol/l | 0.224 [-0.057, 0.505] | 0.102 | 0.12 |

Abbreviations: CI: confidence interval, CVD: cardiovascular disease, HDL: high-density lipoprotein, LDL: low-density lipoprotein. * Reference: tobacco never used.
