# Supplementary material for: Central and local arterial stiffness in White Europeans compared to age-, sex-, and BMI-matched South Asians
Source: PLoS One. 2023 Aug 24;18(8):e0290118. doi: 10.1371/journal.pone.0290118 (PMC10449187; doi:10.1371/journal.pone.0290118)
Supplement: S3 Table — (DOCX) [file pone.0290118.s005.docx]

**S3 Table. Univariable regression coefficients for the associations with pressure-strain elastic modulus Epsilon.**

|  | **Unstandardized B [95% CI]** | **Standardized β** | **p-value** |
| --- | --- | --- | --- |
| South Asian ethnicity | -2.491 [-10.815, 5.833] | -0.038 | 0.56 |
| Age, yrs | 0.867 [0.463, 1.272] | 0.263 | <0.001 |
| Male sex | 1.603 [-6.773, 9.979] | 0.024 | 0.71 |
| Body mass index, kg/m^2^ | 2.086 [0.926, 3.246] | 0.223 | <0.001 |
| Hypertension | 21.738 [7.517, 35.958] | 0.192 | 0.003 |
| History of CVD event | -15.062 [-39.901, 9.777] | -0.111 | 0.23 |
| Former tobacco user * | 0.695 [-10.617, 12.007] | 0.008 | 0.90 |
| Current tobacco user * | 1.297 [-13.471, 16.065] | 0.011 | 0.86 |
| Systolic blood pressure, mmHg | 0.838 [0.650, 1.027] | 0.492 | <0.001 |
| Diastolic blood pressure, mmHg | 0.910 [0.556, 1.264] | 0.311 | <0.001 |
| Total cholesterol, mmol/l | 4.511 [0.417, 8.606] | 0.140 | 0.031 |
| HDL cholesterol, mmol/l | 4.158 [-6.372, 14.688] | 0.051 | 0.44 |
| LDL cholesterol, mmol/l | 4.398 [-0.319, 9.115] | 0.119 | 0.067 |
| Total cholesterol/HDL ratio | 1.968 [-1.789, 5.724] | 0.067 | 0.30 |
| Glucose, mmol/l | 2.333 [-1.567, 6.234] | 0.076 | 0.24 |

Abbreviations: CI: confidence interval, CVD: cardiovascular disease, HDL: high-density lipoprotein, LDL: low-density lipoprotein. * Reference: tobacco never used.
