# Supplementary material for: Central and local arterial stiffness in White Europeans compared to age-, sex-, and BMI-matched South Asians
Source: PLoS One. 2023 Aug 24;18(8):e0290118. doi: 10.1371/journal.pone.0290118 (PMC10449187; doi:10.1371/journal.pone.0290118)
Supplement: S4 Table — (DOCX) [file pone.0290118.s006.docx]

**S4 Table. Pulse transit time and distances for calculating carotid-femoral pulse wave velocity, per cohort.**

|  | **White Europeans n=121** | **South Asians n=121** | **p-value** |
| --- | --- | --- | --- |
| Pulse transit time, ms | 86±20 | 58±14 | <0.001 |
| Sternal notch to left common carotid artery (*i*), mm | 73±13 | 73±14 | 0.68 |
| Sternal notch to top of thigh cuff (*ii*), mm | 751±78 | 656±70 | <0.001 |
| Top of thigh cuff to femoral artery (*iii*), mm | 131±31 | 124±28 | 0.055 |
| Effective path length (*D)*, mm | 553±57 | 459±67 | <0.001 |

Effective path length *D* is calculated as $D=ii-i-iii$. Variables are presented as means ± standard deviations and compared between the cohorts using independent sample t-tests.
